# Genetic Characterization and Pathological Analysis of a Novel Bacterial Pathogen, *Pseudomonas tructae*, in Rainbow Trout (*Oncorhynchus mykiss*)

**DOI:** 10.3390/microorganisms7100432

**Published:** 2019-10-10

**Authors:** Woo Taek Oh, Ji Hyung Kim, Jin Woo Jun, Sib Sankar Giri, Saekil Yun, Hyoun Joong Kim, Sang Guen Kim, Sang Wha Kim, Se Jin Han, Jun Kwon, Se Chang Park

**Affiliations:** 1 Laboratory of Aquatic Biomedicine, College of Veterinary Medicine and Research Institute for Veterinary Science, Seoul National University, Seoul 08826, Korea; mike0202@snu.ac.kr (W.T.O.); giribiotek@gmail.com (S.S.G.); arseidon@snu.ac.kr (S.Y.); hjoong@snu.ac.kr (H.J.K.); imagine0518@snu.ac.kr (S.G.K.); kasey.kim90@gmail.com (S.W.K.); sejin.n@snu.ac.kr (S.J.H.); kjun1002@snu.ac.kr (J.K.); 2 Infectious Disease Research Center, Korea Research Institute of Bioscience and Biotechnology, Daejeon 34141, Korea; kzh81@kribb.re.kr; 3 Department of Aquaculture, Korea National College of Agriculture and Fisheries, Jeonju 54874, Korea; advancewoo@snu.ac.kr

**Keywords:** *pseudomonas tructae*, rainbow trout, genome, bacterial pathogen

## Abstract

*Pseudomonas* species are one of the most prevalent bacterial species globally distributed in forest soil, river water, and human or animal skin. Some species are pathogens or opportunistic pathogens in hospitalized patients, animals, and plants. Various *Pseudomonas* species, including *Pseudomonas* *putida*, *P. plecoglossicida*, *P. aeruginosa*, and *P. fluorescens*, are known fish pathogens; *P. fluorescens* and *P. putida* cause severe losses in rainbow trout farming. Therefore, we investigated and isolated the pathogen that is responsible for mortality in a rainbow trout farm in Korea. The isolated bacterium was a strain of *P. tructae*, which was recently classified in the *P. putida* group. We performed taxonomical analysis of the bacteria in our previous study. In this study, we investigated the pathogenicity and clinical symptoms of *P. tructae* and analyzed its genomic characteristics. The pathogenicity of the strain was tested via challenge experiments in healthy rainbow trout and histopathologic analysis of the infected fish. Genome sequence was analyzed to identify the bacterial genes that are involved in antibiotic resistance and virulence. This is the first study reporting *P. tructae* as an emerging pathogen that is responsible for mortality in rainbow trout fisheries and providing the genome sequence of *P. tructae*.

## 1. Introduction

*Pseudomonas* species are gram-negative, rod-shaped bacteria dwelling in environments, such as soil, water, and animal skin [1,2]. Generally, these bacteria grow well in aerobic conditions and thrive in various environmental conditions, including marine habitats, and they play different roles, such as in the spoilage of milk products [3,4]. More than 70 different *Pseudomonas* species have been identified, and they are classified into the following seven genogroups [5]: *Pseudomonas aeruginosa, P. chlororaphis, P. fluorescens, P. pertucinogena, P. putida, P. stutzeri*, and *P. syringae*. The pathogenicity of the *Pseudomonas* species has been reported in animals, plants, and immune-compromised hospitalized patients [6]. One of the representative species, *P. syringae*, is a plant pathogen that infects different commercial plants, thereby resulting in huge economic losses [7]. *P. aeruginosa* is a pathogenic bacterium that is commonly responsible for nosocomial infections in immune-deficient patients [8]; it has been extensively studied due to its defense mechanism against diverse antibiotics and multi-antibiotic resistance [9]. In addition, *P. putida* is considered as an opportunistic human pathogen that causes diverse clinical infections, including eye infection, burn site infection, and wound and skin infections [10,11]. Most *Pseudomonas* spp. are naturally resistant to several antibiotics, such as beta-lactams, owing to the presence of efflux pumps [12], which play an important role in antibiotic resistance that are involved in the efflux of toxic molecules from the bacterial cells. *P. oryzihabitans* and *P. plecoglossicida* are well-known animal pathogens that cause sepsis and bacteremia in warm-blooded and aquatic animals [13,14]. Many *Pseudomonas* spp. are considered to be opportunistic pathogens, even though they exist normally under natural environmental conditions.

Diverse *Pseudomonas* spp. are also pathogenic to fish, causing severe economic losses in the aquaculture industry. The pathogenicity of Pseudomonads has been reported in various fish species. *P. putida* has been reported to be pathogenic in fishes, such as ayu (*Plecoglossus altivelis altivelis*), rainbow trout (*Oncorhynchus mykiss*), and yellow tail (*Seriola quinqueradiata*) [10,15,16]. In addition, the pathogenicity of *P. aeruginosa*, *P. fluorescens*, *P. anguilliseptica*, and *P. plecoglossicida* has been reported in diverse fish species [17,18]. These species are responsible for considerable economic losses in the cultivation of tilapia (*Oreochromis mossambicus*) [19], rainbow trout [20], eel (*Anguilla japonica*), and ayu, respectively [15]. Of these bacteria, *P. fluorescens* and *P. putida* are known as major pathogens in rainbow trout fisheries [20].

In this study, we investigated the pathogen that is responsible for mortality in a rainbow trout farm in the Chungbuk Province of Korea. Using histopathology and genetic analysis of the bacterial strain, we confirmed the presence of *P. tructae*, a recently identified bacterium, belonging to the *P. putida* group, on the basis of its phenotypical and phylogenetic analysis [21]. This was achieved through phylogenetic analysis, while using the sequences of three house-keeping genes and 16S rRNA gene. Additionally, the phenotypical differentiation and genome to genome distance values were measured for its confirmation as a novel species. As far as we know, this is the first report detailing the pathogenicity of *P. tructae* infection, which leads to mortality and economic losses in rainbow trout fisheries in Korea. In March 2018, a rainbow trout farm located in the Chungbuk Province in Korea requested that we investigate and identify the cause of mortality on the farm. The farm raised 100,000 rainbow trout fry in eight water tanks; each water tank contained 5,000 L of water, and the facility used a closed circular water system that was supplemented with ground water. The water temperature was 12–13 °C and the dissolved oxygen concentration was estimated as 8.1 ± 0.3 mg/L. The natural death rate was below 1%, but the mortality rate surged to 15% during the disease outbreak. Diseased trout showed clinical signs of lethargic activity, skin darkening, appetite loss, and erratic swimming behavior, including floating on the shallow side of the water tank.

## 2. Materials and Methods

### 2.1. Fish Sampling and Postmortem Examination

The size of the affected fish was approximately 12 ± 3 g. Ten fish showing such severe clinical signs were selected, preserved at 4 °C, and then sent to Laboratory of Aquatic Biomedicine, Seoul National University, for the diagnosis.

In the laboratory, the fish were examined for any infection that might have caused the observed mortality. External lesions were first visually examined. To detect parasitic and fungal infections, the gills and the caudal and dorsal fins of each fish were swabbed and smeared on a glass slide. The smeared glass slides were observed under a light microscope. Internal organs, including kidney, liver, and spleen, were collected for RNA extraction to identify viral infection. Tissues were pooled in 300 µL phosphate-buffered saline (PBS) and homogenized while using a tissue homogenizer. RNA was extracted from the homogenized solution using the Patho Gene-spin kit (iNtRON Biotechnology, Daejeon, Korea) according to the manufacturer’s protocol. The cDNA was synthesized from the extracted RNA while using Prime Script 1st strand cDNA synthesis kit (TaKaRa, Shiga, Japan), as per the manufacturer’s protocol. As infectious hematopoietic necrosis virus (IHNV), infectious pancreatic necrosis virus (IPNV), and viral hemorrhagic septicemia virus (VHSV) are the most common viruses infecting salmonids, the presence of these viruses was investigated while using PCR with specific diagnosis primers, as described previously [22,23]. For bacterial infections, the kidney, liver, and spleen homogenates were individually streaked on tryptic soy agar (TSA; BD Difco, Sparks, MD, USA) and brain heart infusion (BHI) agar (BD Difco, Sparks MD, USA). The streaked plates were incubated for 48 h at 20 °C and 25 °C, respectively, under aerobic conditions.

### 2.2. Bacterial Isolation

The streaked plates were examined every 12 h for bacterial growth. The colonies that formed on both types of plates were similar; therefore, the single largest colony was selected for pure isolation. It was sub cultured on the agar plates under the same growth conditions. Pure bacterial colonies were selected for the identification and was preserved at −80 °C in 25% glycerol for use in the challenge trial. For bacteria identification, the detailed analytical results have been specified in a previous study [21]. Although *P. tructae* was identified as a member of the *P. putida* group, no study has been performed to assess the pathogenicity of this bacterium. Hence, a challenge trial was carried out to confirm its pathogenic role.

### 2.3. Challenge Trial

Rainbow trout (average weight, 15 g) were purchased from another trout farm located in Kangwon Province, Korea. The purchased trout were reared in a water tank for two weeks for stabilization before the challenge trial and to check whether the individuals carried any disease. The experiment was carried out at the Department of Aquaculture, Korea National College of Agriculture and Fisheries, under strict ethical guidelines of fish handling. The bacteria that were used for the challenge trial were prepared in tryptic soy broth (TSB) incubated at 25 °C for 24 h in a shaking incubator. Before the trial, the bacteria suspensions were diluted on the basis of optical density measured while using a SmartSpec™ 3000 spectrophotometer (Bio-Rad, Hercules, CA, USA). The suspensions were centrifuged at 8000× *g* for 5 min, and the bacterial pellet was washed with PBS for clear result. The bacterial dilutions used for the challenge were 6 × 10 ^7^, 6 × 10 ^6^, 6 × 10 ^5^, and 6 × 10 ^4^ CFU of bacteria suspended in 0.1 mL PBS. Each experimental group consisted of 10 fish, and each fish was administered intraperitoneal injections of the indicated concentration of bacteria; the control fish were injected with the same volume of PBS. All of the infection experiments were simultaneously performed in triplicate, and the infected fish groups were reared in different 120L water tanks. The temperature of the water was maintained at 13 °C, identical to the temperature maintained in the trout farm where the disease outbreak occurred. Each group was individually aerated and observed twice daily for 15 days to determine the mortality rate. During this observation, fish showing the clinical symptoms of lethargic movement, skin darkening, and erratic swimming movements were selected for histopathological analysis and the re-isolation of the bacteria. The re-isolation procedure was performed as described in Section 2.2.

### 2.4. Histopathological Analysis

Selected fish from the challenge trial were euthanized and post mortem examination was performed to investigate the bacterial infection. The internal organs, including kidney, liver, and spleen, were excised from the fish. The tissue samples were fixed in 10% neutral-buffered formalin. The fixed tissues were sliced, dehydrated while using ethanol, and embedded in paraffin blocks, which were then sectioned. The tissue sections were stained with hematoxylin and eosin and analyzed using light microscopy and digitally scanned by Xenos Inc. (Seoul, Korea).

### 2.5. Antibiotic Susceptibility Test

The bacteria re-isolated from the diseased fish were tested for antibiotic susceptibility. The test was performed while using the disk diffusion method and the estimation of minimum inhibitory concentrations (MICs). *Pseudomonas* spp. are known to have high resistance to beta-lactam antibiotics. Therefore, the bacterial strain was cultured on Mueller-Hinton agar (BD Difco) and then tested for antibiotic susceptibility while using the disk diffusion method. The results were analyzed after 24 h, as described in the Clinical & Laboratory Standards Institute (CLSI) M100 guidelines [24]. The following antibiotics were used in the study: amoxicillin-clavulanate, ampicillin-sulbactam, piperacillin-tazobactam, cefazolin, cefepime, cefotaxime, aztreonam, cefoxitin, ceftazidime, imipenem, meropenem, amikacin, tetracycline, ciprofloxacin, levofloxacin, trimethoprim-sulfamethoxazole, chloramphenicol, ampicillin, amoxicillin, and gentamycin. The same procedure was performed to determine the MICs of the antibiotics, which were selected while using the conventional macrodilution technique in Mueller-Hinton agar with calibrated concentrations of divalent cations [24]. The following antibiotics were tested: amoxicillin-clavulanate, cefotaxime, imipenem, meropenem, amikacin, tetracycline, ciprofloxacin, levofloxacin, ampicillin, amoxicillin, and gentamycin. The results were classified as resistant, intermediate, or susceptible, according to the CLSI breakpoints.

### 2.6. Genome Characterization

For complete genome analysis, the genome of the isolated bacterial strain (SNU WT1) was sequenced by Macrogen Inc. (Daejeon, Korea) while using the hybrid approach with a PacBio RS II (Pacific Biosciences, USA) and HiSeq 2000 instrument (Illumina, USA). The complete genome sequence of the strain has been registered in GenBank (Accession number: CP035952). As *P. tructae* was recently classified as a *Pseudomonas* species, the genome was analyzed for the presence of genes that were related to antibiotic resistance and virulence. The presence of potential virulence genes and antimicrobial resistance genes was identified by searching the virulence factor database (http://www.mgc.ac.cn/VFs/) and the ARG-ANNOT database (http://en.mediterranee-infection.com/articlc.php?laref=283&titre=arg-annot-), respectively. The presence of various related genes was determined while using the local BLAST option in the BioEdit software to compare the genome sequence with the sequence of genes present in other *Pseudomonas* spp. The maximum expectation value for the searches was fixed at 0.0001.

## 3. Results and Discussion

### 3.1. Postmortem Examination of the Diseased Fish

As the mortality rate of the farm surged to 15%, the diseased trout showing clinical signs of lethargic activity, darkening of the skin, and loss of appetite were collected for the examination. Ten diseased fish from the rainbow trout farm were collected for the post mortem examination. The fish did not show any external clinical signs such as anemic appearance on gills and damage on the fins when visually inspected. The gills and caudal and the dorsal fins showed a negative result on fungal and parasitic infections when observed under light microscope. The internal organs did not show specific lesions, but slight swelling lesions were observed in the kidneys of the diseased fish. The tissues, including liver, kidney, and spleen, were collected for the examination of any viral and bacterial infections. The RNA extraction was performed under manufacturer’s protocol and additionally, the PCR-based tests for detection of IHNV, IPNV, and VHSV were negative for all of the fish. After 24 h of incubation on two types of agar plates at 20 °C and 25 °C, a uniformly shaped reddish colony with a diameter of 1–2 mm appeared on both agar plates [21]. A single colony was selected for pure isolation of the bacteria, and the colony grew well on TSA, under the same growth conditions.

### 3.2. Pathogenicity of the Bacterial Strain

**Figure 1:**
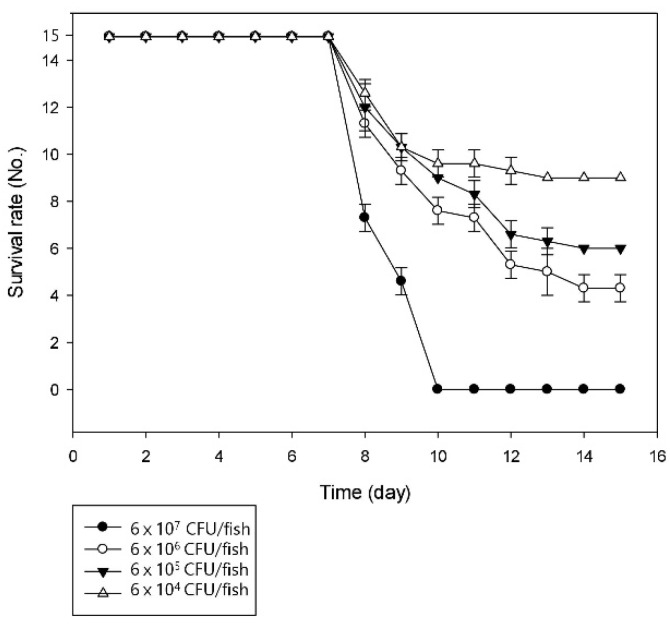
Challenge trial results indicating the average number of surviving fish per group at 15 days post infection.

Similar conditions were created for artificial infection to prove the pathogenicity of the bacterial strain SNU WT1 in rainbow trout. The infected fish showed signs of lethargic activity, skin darkening, and erratic swimming motions, and finally resulted in the death of the fish. LD_50_ of the strain was calculated after observation for 15 days (Figure 1).

The LD_50_ value was estimated to be 1.5 × 10 ^5^ CFU/fish, which was clearly virulent for trout. The estimated value was lower than that reported in a previous study on *P. putdia*, which was 5 × 10^6^ CFU/fish [10]. Mortality or similar clinical signs were not observed in any of the fish in the control group. The overall survival rate of fish was 0% when the titer was greater than 6 × 10^7^ CFU/fish. The onset of mortality was observed at seven days post infection, regardless of the dose of the injected bacteria and the survival rate of the fish more rapidly decreased in higher challenged titers. Postmortem examination of the diseased fish revealed kidney swelling and liver congestion. Bacteria were isolated from the kidneys of the 10 dead fish from the challenge trial; the same bacterium was isolated from every fish.

### 3.3. Histopathological Analysis

**Figure 2:**
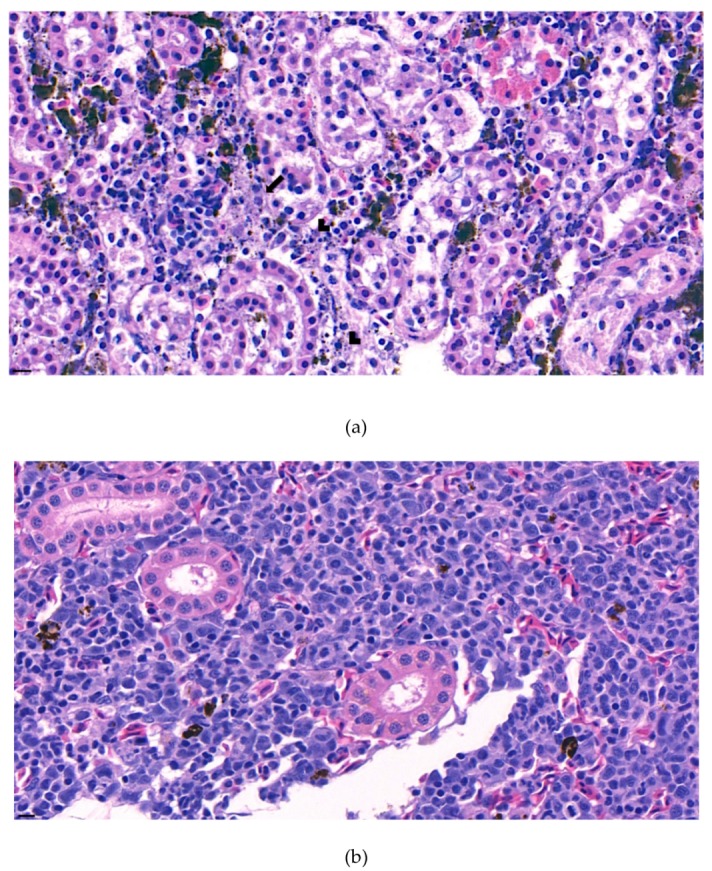
(**a**). Representative image of hematoxylin and eosin-stained tissue section showing degenerated tubules surrounded by lymphocytes and macrophages in the kidney of the diseased fish. Signs of phagocytized rod-shaped bacterium and cellular debris can also be observed. (arrow: phagocytized rod-shaped bacterium, arrow head: cellular debris) (**b**). Negative fish group kidney image stained with hematoxylin and eosin. (Bar indicating 10 µm).

**Figure 3:**
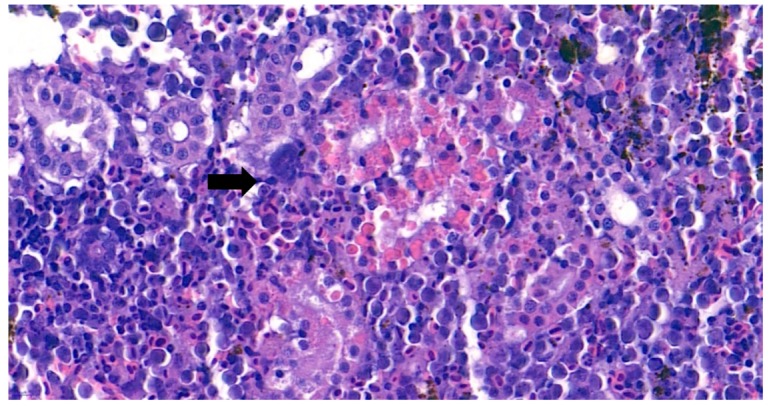
Representative image of hematoxylin and eosin-stained tissue section showing hyaline droplet accumulation in the tubular epithelium in the kidney of the diseased fish. Bacteremia can be observed in the area indicated by the arrow. (Bar indicating 10 µm).

The internal lesions of the moribund fish were concentrated on the liver and kidney. Therefore, the spleen, kidney, and liver of five diseased fish were histopathologically analyzed. Significant tubular degeneration (Figure 2) and hyaline droplet accumulation in tubular epithelium (Figure 3) was observed in the infected posterior kidneys.

**Figure 4:**
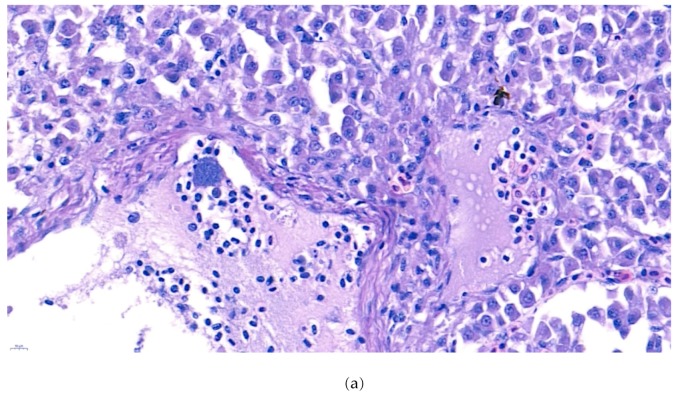

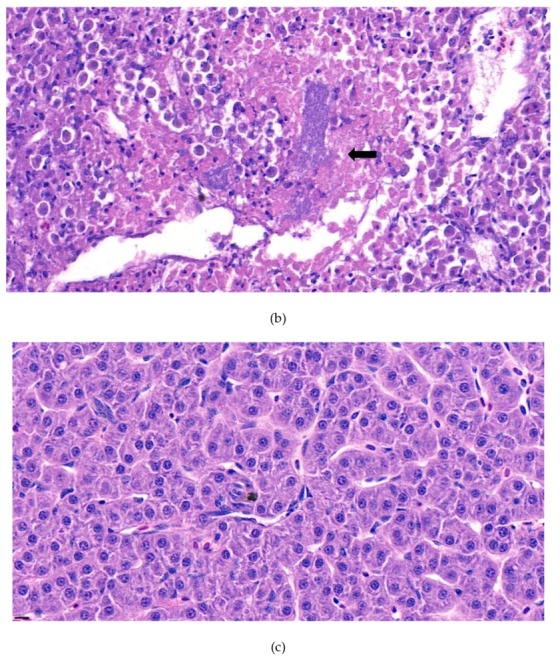
(**a**). Representative image of hematoxylin and eosin-stained tissue section showing focal necrotizing areas in the liver of the diseased fish. Cellular vacuolation can be observed around the lesion. (Bar indicating 10 µm). (**b**). Representative image of hematoxylin and eosin-stained tissue section showing infiltration of macrophages and lymphocytes with signs of bacteremia (arrow) in the liver of the diseased fish. 4 (**c**). Negative fish group liver image stained with hematoxylin and eosin. (Bar indicating 10 µm).

Furthermore, the infiltration of diverse immunocytes, including lymphocytes and macrophages, was observed surrounding the tubule (Figure 2). In addition, signs of bacteremia along the degenerated tubule (Figure 3) were observed with cellular debris surrounding the tubules (Figure 2). Similar signs were also observed in the liver of the diseased fish. A few necrotizing areas with infiltration of macrophages and lymphocytes and vacuolation of hepatic cells was observed (Figure 4a,b).

Similar severity of lesion formation was not observed in the spleen, which suggested the liver and kidney as the target organs of the bacteria. Signs of bacteremia and cellular degeneration were observed in the liver and kidney, but not in the spleen. Therefore, the cause of the mortality of fish can be speculated as bacteremia.

### 3.4. Antibiotic Susceptibility Result

As *Pseudomonas* spp. are known to be resistant to diverse antibiotics, especially beta-lactams, the isolated strain was expected to show multi-antibiotic resistance. Antibiotic susceptibility analysis using the disk diffusion method revealed that the strain was resistant to most antibiotics, except tetracycline and ciprofloxacin. Similarly, MIC determination showed that the strain was susceptible to only two antibiotics (Table 1). These results, especially the resistance to ampicillin-clavulanate, piperacillin-tazobactam, and levofloxacin, indicate that the strain shows characteristics that are similar to those of other *Pseudomonas* spp. [10,12,25]. Furthermore, the susceptibility to tetracycline suggests that this antibiotic can be used for the treatment of this bacterial infection.

### 3.5. Genome Features of Strain SNU WT1

The complete genome of the strain SNU WT1 had a total length of 5,685,196 bp with 61.8% G+C content. The genome contained 74 tRNA-coding and 22 rRNA-coding genes and 5,083 coding sequences (CDSs). Ten gene fragments were identified as potential antibiotic resistance genes (Supplementary Table S2). In brief, the genome of the strain SNU WT1 contained genes for resistance to the following antibiotics: fluoroquinolones, tetracyclines, beta-lactams, and macrolide-lincosamide-streptogramin. Coupled with the resistance to fluoroquinolones and beta-lactams that were observed in the antibiotic susceptibility test, there are indications that these genes might contribute to antibiotic resistance in the SNU WT1 strain. However, as the strain was highly susceptible to tetracycline, the identified potential genes (listed in Supplementary Table S2) might not be involved in tetracycline resistance. This could have resulted from the alignment length (only 120 bp), which could have resulted in the expression of the genes, being invalid; alternatively, the fragment, which is important for the gene expression, could have been deleted, thus rendering the remaining part useless. The virulence factor search showed many gene fragments as potential genes that are involved in the virulence of the strain. Most of them were closely related to the virulence factors of *P. putida* and *P. aeruginosa* strains, such as flagellar biosynthesis protein, phosphomannomutase, flagellar motor switch protein, and type IV pili twitching motility-related proteins (Supplementary Table S1). The genome comparison with virulence factor database already annotated in previous researches using BLAST search showed that the potential virulence of the strain might be similar to *P. putida* infection in rainbow trout

### 3.6. General Information about Strain SNU WT1 in This Study

In our study, we focused on the pathogenicity of *P. tructae* strain SNU WT1, a bacterial species classified into the *P. putida* group. Challenge trial and histopathologic analysis were performed to determine the pathogenicity of the strain. The previously reported LD_50_ value for *P. putida* in rainbow trout was higher than 5 × 10 ^6^ CFU/fish [10]. Our study revealed a lower LD_50_ value, which indicating that the strain, SNU WT1, might be more virulent than *P. putida*. Furthermore, unlike *P. putida*, which causes ulcerative disease in rainbow trout, the strain. SNU WT1 induced bacteremia on liver and kidney causing death. The histopathology results showed that tubular degeneration and signs of bacteremia in both liver and kidney are among the main clinical symptoms associated with *P. tructae* strain SNU WT1 infection. Similar to other known fish pathogenic bacteria such as *Aeromonas* sp., *Vibrio* sp., and *Edwardsiella* sp., this bacterial strain caused septicemia-induced death [26]. Septicemia is also a common clinical symptom of *Pseudomonas* spp. infection in fish; for example, *P. plecoglossicida* induced hemorrhagic septicemia in ayu [14] and *P. anguilliseptica* induced similar symptoms in eel [17]. Therefore, *P. tructae* might be considered to be a virulent salmonid pathogen.

Antibiotic susceptibility analysis revealed the multi-antibiotic resistance of *P. tructae* strain SNU WT1, which was similar to that observed in other *Pseudomonas* spp. This indicates that the *P. tructae* strain SNU WT1 may be a considerable threat in rainbow trout farming. However, it was susceptible to tetracycline, one of the most commonly used antibiotics in aquaculture; therefore, tetracycline treatment might be currently effective for treating infections with *P. tructae* strain SNU WT1. Nevertheless, as *Pseudomonas* spp. have various abilities to gain antibiotic resistance [25], this antibiotic might not be effective in the near future. In addition, genome analysis of strain SNU WT1 indicated the presence of diverse antibiotic resistance genes and virulence genes. As this is the first study providing the genomic information of *P. tructae*, the data provided here may serve as a foundation for understanding the similarity of this strain with the diverse *Pseudomonas* species, in order to understand its ability to gain different antibiotic resistance genes and to identify the specific role of each gene.

## 4. Conclusions

In conclusion, this study reports *P. tructae* as a virulent *Pseudomonas* species that can affect rainbow trout fisheries and cause economic losses through mortality. Although the distribution of this species is presently limited to Korea, owing to its pathogenicity, further studies are needed to evaluate its epidemiological distribution. Moreover, as *P. tructae* was recently classified as a species in the *P. putida* group, research reporting the outbreak of its infection in aquaculture will be help to address an important concern in rainbow trout farming.

## Figures and Tables

**Table 1:** Antibiotic susceptibility analysis performed while using the disk diffusion method and the determination of minimum inhibitory concentrations (MICs).

Antibiotic Name	Results of Disk Diffusion Assays	Inhibition Zone Diameter (mm)	Results of MIC Assays	MIC (µg/mL)
Amoxicillin-clavulanate	R	0	R	256
Ampicillin-sulbactam	R	0	−	−
Piperacillin-tazobactam	R	26	−	−
Cefazolin	R	0	−	−
Cefepime	R	28	−	−
Cefotaxime	R	12	R	32
Aztreonam	R	10	−	−
Cefoxitin	R	0	−	−
Ceftazidime	I	20	−	−
Imipenem	R	22	R	4
Meropenem	R	18	R	8
Amikacin	R	24	R	64
Tetracycline	S	24	S	4
Ciprofloxacin	S	42	S	0.06
Levofloxacin	R	32	I	4
Trimethoprim-sulfamethoxazole	I	16	−	−
Chloramphenicol	R	0	−	−
Ampicillin	R	6	R	256
Amoxycillin	R	10	R	256
Gentamycin	R	9	R	16

R: resistant, I: intermediate, S: susceptible.

## Supplementary Materials

The following are available online at https://www.mdpi.com/2076-2607/7/10/432/s1.
