# Supplementary material for: Genetic Characterization and Pathological Analysis of a Novel Bacterial Pathogen, Pseudomonas tructae, in Rainbow Trout (Oncorhynchus mykiss)
Source: Microorganisms. 2019 Oct 10;7(10):432. doi: 10.3390/microorganisms7100432 (PMC6843698; doi:10.3390/microorganisms7100432)
Supplement: Supplementary file 1 [file microorganisms-07-00432-s001.pdf]

Query id	Database id	% Identity	Alignment length	Gap	QSS*	QSE**	DSS***	DSE****	e-value	Bit score
CP035952.1	VFG014597(gi:148546762) (flhA) flagellar biosynthesis protein FlhA [Flagella (CVF521)] [Pseudomonas putida F1]	86.09%	2186	7	2006681	2008865	41	2220	0	2565 bits
CP035952.1	VFG015761(gi:170719370) (algC) Phosphomannomutase [Alginate biosynthesis (CVF522)] [Pseudomonas putida W619]	85.94%	1401	3	184539	185939	1	1398	0	1637 bits
CP035952.1	VFG015712(gi:167034908) (flhA) flagellar biosynthesis protein FlhA [Flagella (CVF521)] [Pseudomonas putida GB-1]	87.14%	2100	0	2006763	2008862	1	2100	0	2571 bits
CP035952.1	VFG014050(gi:104783970) (pilJ) type IV pili methyl-accepting chemotaxis transducer PilJ [Type IV pili twitching motility related proteins (CVF519)] [Pseudomonas entomophila L48]	86.01%	2001	4	757553	759551	56	2054	0	2342 bits
CP035952.1	VFG015479(gi:70733374) (PFL_6091) hypothetical protein [Hcp secretion island-1 encoded type VI secretion system (H-T6SS) (CVF535)] [Pseudomonas fluorescens Pf-5]	83.36%	1863	6	5659439	5661298	1	1860	0	1959 bits
CP035952.1	VFG015075(gi:104780548) (gacS) GacS sensor kinase protein [GacS/GacA two-component system (CVF529)] [Pseudomonas entomophila L48]	83.32%	2751	15	4582623	4585364	13	2757	0	2870 bits
CP035952.1	VFG014414(gi:26991058) (fliF) flagellar M-ring protein [Flagella (CVF521)] [Pseudomonas putida KT2440]	85.75%	1782	3	1991797	1993578	1	1779	0	2067 bits
CP035952.1	VFG014765(gi:148549640) (alg8) hypothetical protein [Alginate biosynthesis (CVF522)] [Pseudomonas putida F1]	88.14%	1492	2	5021408	5022898	1	1491	0	1886 bits
CP035952.1	VFG014366(gi:77457759) (fleQ) Sigma-54 Specific Transcriptional Regulator, Fis family [Flagella (CVF521)] [Pseudomonas fluorescens Pf0-1]	86.79%	1476	0	1987067	1988542	1	1476	0	1783 bits
CP035952.1	VFG015449(gi:70733371) (PFL_6088) hypothetical protein [Hcp secretion island-1 encoded type VI secretion system (H-T6SS) (CVF535)] [Pseudomonas fluorescens Pf-5]	92.16%	1492	0	5656755	5658246	1	1492	0	2164 bits
CP035952.1	VFG020007(gi:218888829) (PLES_00851) hypothetical protein [Hcp secretion island-1 encoded type VI secretion system (H-T6SS) (CVF535)] [Pseudomonas aeruginosa LESB58]	89.07%	1473	5	5656777	5658248	1	1497	0	1927 bits
CP035952.1	VFG002070(gb|NP_248774) (hsiC1/vipB) type VI secretion system tubule-forming protein VipB [HSI-I (VF0334)] [Pseudomonas aeruginosa PAO1]	89.00%	1473	5	5656777	5658248	1	1497	0	1922 bits
CP035952.1	VFG014890(gi:77457176) (algA) Mannose-1-phosphate guanylyltransferase/mannose-6-phosphate isomerase [Alginate biosynthesis (CVF522)] [Pseudomonas fluorescens Pf0-1]	86.50%	1452	0	5034673	5036124	1	1452	0	1736 bits
CP035952.1	VFG002076(gb|NP_248780) (clpV1) type VI secretion system AAA+ family ATPase [HSI-I (VF0334)] [Pseudomonas aeruginosa PAO1]	83.54%	2704	34	5662307	5664978	1	2702	0	2874 bits
CP035952.1	VFG015492(gi:116053810) (clpV1) putative ClpA/B-type chaperone [Hcp secretion island-1 encoded type VI secretion system (H-T6SS) (CVF535)] [Pseudomonas aeruginosa UCBPP-PA14]	83.51%	2705	36	5662307	5664978	1	2702	0	2874 bits
CP035952.1	VFG015369(gi:70733358) (PFL_6075) Domain of unknown function (DUF323) family [Hcp secretion island-1 encoded type VI secretion system (H-T6SS) (CVF535)] [Pseudomonas fluorescens Pf-5]	84.36%	1618	0	5640337	5638720	1	1694	0	1778 bits
CP035952.1	VFG014754(gi:104783501) (algD) GDP-mannose 6-dehydrogenase [Alginate biosynthesis (CVF522)] [Pseudomonas entomophila L48]	89.22%	1317	0	5020035	5021351	1	1317	0	1736 bits
CP035952.1	VFG014455(gi:104782818) (fliI) flagellum-specific ATP synthase [Flagella (CVF521)] [Pseudomonas entomophila L48]	86.13%	1327	0	1995426	1996752	1	1327	0	1564 bits
CP035952.1	VFG015752(gi:167035560) (algI) membrane bound O-acyl transferase MBOAT family protein [Alginate biosynthesis (CVF522)] [Pseudomonas putida GB-1]	89.23%	1458	3	5031355	5032809	1	1458	0	1920 bits
CP035952.1	VFG042736(gi:15599658) (rpoN) RNA polymerase factor sigma-54 [type IV pili (AI097)] [Pseudomonas aeruginosa PAO1]	84.45%	1498	12	1412643	1411152	1	1492	0	1636 bits
CP035952.1	VFG014448(gi:152986739) (fliI) flagellar protein export ATPase FliI [Flagella (CVF521)] [Pseudomonas aeruginosa PA7]	82.87%	1343	4	1995426	1996766	1	1341	0	1376 bits
CP035952.1	VFG014993(gi:70728315) (algW) peptidase, S1C (protease Do) subfamily [Alginate regulation (CVF523)] [Pseudomonas fluorescens Pf-5]	87.13%	1158	3	4997675	4998832	1	1155	0	1416 bits
CP035952.1	VFG014990(gi:26988036) (algW) HtrA-like protease AlgW [Alginate regulation (CVF523)] [Pseudomonas putida KT2440]	86.01%	1158	1	4997661	4998818	1	1192	0	1355 bits

CP035952.1	VFG020019(gi:229593390) (clpV1) chaperone [Hcp secretion island-1 encoded type VI secretion system (H-T6SS) (CVF535)] [Pseudomonas fluorescens SBW25]	86.54%	2675	21	5662307	5664978	1	2657	0	3185 bits
CP035952.1	VFG001242(gb|NP_249775) (flgI) flagellar P-ring protein precursor FlgI [Flagella (VF0273)] [Pseudomonas aeruginosa PAO1]	84.13%	1109	8	1964495	1965599	1	1110	0	1196 bits
CP035952.1	VFG000123(gb|NP_252231) (alg8) alginate-c5-mannuronan-epimerase AlgG [Alginate (VF0091)] [Pseudomonas aeruginosa PAO1]	84.33%	1436	6	5021451	5022883	1	1470	0	1572 bits
CP035952.1	VFG015686(gi:167034924) (fliG) flagellar motor switch protein FliG [Flagella (CVF521)] [Pseudomonas putida GB-1]	88.73%	1020	0	1993571	1994590	1	1020	0	1322 bits
CP035952.1	VFG016056(gi:77460153) (pvdH) Diaminobutyrate-2-oxoglutarate transaminase [Pyoverdine (CVF551)] [Pseudomonas fluorescens Pf0-1]	81.75%	1293	8	2137438	2138726	1	1346	0	1262 bits
CP035952.1	VFG015826(gi:167034804) (pvdL) amino acid adenylation domain protein [Pyoverdine (CVF551)] [Pseudomonas putida GB-1]	78.13%	13027	169	2118223	2131163	1	12944	0	10400 bits

Supplementary table 1. Genome analysis result of strain SNU WT1 based on sequence database of virulence factors in other Pseudomonas species.

Chromosome	Database id	Annotation	% Identity	Alignment length	Gap	QSS	QSE	DSS	DSE	e-value	Bit score
CP035952.1	(Flq)OqxBgb:EU370913:47851-51003:3153	Integral membrane protein. component of RND-type multidrug efflux pump that confers resistance to olaquindox	75.24%	206	0	2829688	2829893	1054	1259	3.00E-20	99.0 bits
CP035952.1	(Tet)otrA:X53401:349-2341:1992	oxytetracycline resistance	74.17%	120	2	4152345	4152227	508	626	9.00E-12	70.7 bits
CP035952.1	(Bla)CMY-19:AB194410:1-1146:1146	beta-lactamase/D-alanine carboxypeptidase	65.29%	1020	46	3689420	3690422	144	1134	1.00E-52	205 bits
CP035952.1	(Bla)CMY-11:AF381626:254-1402:1149	beta-lactamase/D-alanine carboxypeptidase	65.23%	1018	42	3689420	3690422	144	1134	3.00E-53	206 bits
CP035952.1	(Bla)FOX-10:JX049131:1-1149:1149	beta-lactamase/D-alanine carboxypeptidase	65.16%	1019	39	3689420	3690420	135	1132	2.00E-43	174 bits
CP035952.1	(Flq)OqxBgb:EU370913:47851-51003:3153	integral membrane protein, component of RND-type multidrug efflux pump that	71.22%	1595	19	2808203	2806617	1	1584	7.00E-86	762 bits

		confers resistance to olaquindox									
CP035952.1	(Bla)FOX-8:HM565917:1-1149:1149	beta-lactams hydrolysis	65.44%	975	37	3689463	3690420	178	1132	4.00E-46	183 bits
CP035952.1	(MLS)TlrC:M57437:277-1923:1647	Tylosin resistance protein (tlrC) gene	74.17%	120	2	4152345	4152227	508	626	9.00E-12	70.7 bits
CP035952.1	(MLS)SrmB:X63451:558-2210:1653	ATPase components of ABC transporters with duplicated ATPase domains	70.99%	131	0	4151441	4151311	557	687	1.00E-10	66.2 bits
CP035952.1	(Bla)OCH-5:AJ295343:1-1173:1173	beta-lactams hydrolysis	66.98%	630	18	3689397	3690017	127	747	6.00E-38	157 bits

QSS: query sequence start, QSE: query sequence end, DSS: database sequence start, and DSE: database sequence end

Supplementary Table 2. Genome sequence analysis of SNU WT1 strain for detecting potential virulence factors and antibiotic resistance genes.
